# Translating Research on Evolutionary Transitions Into the Teaching of Hierarchical Complexity in University Biology Courses

**DOI:** 10.1002/ece3.72267

**Published:** 2025-11-02

**Authors:** SoRi La, Zachariah I. Grochau‐Wright, Joshua S. Hoskinson, Dinah R. Davison, Richard E. Michod

**Affiliations:** ^1^ Department of Ecology and Evolutionary Biology University of Arizona Tucson Arizona USA; ^2^ Department of Biology Colorado State University Fort Collins Colorado USA; ^3^ School of Life Sciences Arizona State University Tempe Arizona USA; ^4^ Division of Biology Kansas State University Manhattan Kansas USA

**Keywords:** evolutionary transitions in individuality, hierarchy of life, major evolutionary transitions, teaching biological complexity, teaching evolution, vision and change

## Abstract

The hierarchy of life is the organizing framework for biology. An analysis of commonly used U.S. undergraduate biology textbooks revealed that while introductory college biology textbooks teach this framework, its origin and evolution are not taught. In the next tier of college courses devoted to evolution, the hierarchy of life is given little or no emphasis as an organizing framework. Hierarchical structure was not present at the origin of life; it evolved, yet biology courses are not teaching how and why it evolved. Here, we use the theory of evolutionary transitions in individuality (ETIs) to teach the evolution of nested hierarchical structure. ETI theory explains how groups of cooperating individuals evolved into new kinds of evolutionary individuals that make up the hierarchy of life. We demonstrate how incorporating ETI theory into college courses unites common subjects and learning goals while aligning with “Vision and Change,” a common framework for designing effective and relevant university biology curricula. Since ETI theory is based on the same social principles students encounter in their lives, we expect this approach to be both engaging and intuitive to students. Teaching ETI theory addresses a major gap in biology instruction: the disconnect between using the hierarchy of life as an organizing framework, but not teaching how and why it evolved.

## Graphical abstract

Introductory biology courses use the hierarchy of life as an organizing framework but rarely teach how and why it evolved. We propose incorporating ETI theory into college curricula to address this gap, demonstrating how groups of cooperating individuals became new evolutionary units that make up the hierarchy of life. This approach unifies key evolution concepts, aligns with the “Vision and Change” framework, and leverages social principles to enhance student engagement and understanding.

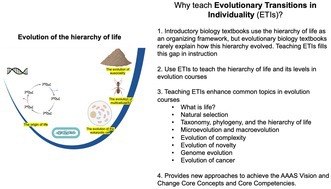

## 1 Introduction

Nested hierarchical structure is one of life's most recognizable features and serves as an organizing framework for biological diversity and complexity. Genes are nested in genomes, genomes nested in cells, bacterial cells nested in eukaryotic cells, cells nested in multicellular organisms, and organisms nested in eusocial societies. This hierarchical organization was not present at the origin of life; it evolved, and students should understand how and why it evolved.

Most biology curricula in K‐12 (Hoskinson et al. 2024) and university levels (see below) assume hierarchical organization and use the hierarchy of life as an organizing framework for the teaching and learning of biology. Unfortunately, there are limited instructional resources available to teachers on how and why hierarchical organization evolved. This results in an apparent gap in instruction, a gap between what is assumed to be the organizing framework for biology and what evolutionary biology can explain. This gap in instruction may give the impression that evolution cannot explain one of the most basic and familiar features of life, hierarchical organization, and could lead students to adopt non‐scientific explanations of biological complexity (Behe 2006; Dembski 2005). The main goal of our paper is to show how this gap can be addressed at the college level by using the theoretical framework of evolutionary transitions in individuality (ETI theory) to teach the origin and evolution of hierarchical organization.

ETI theory explains how groups of cooperating individuals evolved into the new kinds of evolutionary individuals that constitute the levels in the hierarchy of life (e.g., the transition from groups of cells to multicellular organisms) (Michod et al. 2022). These rare but repeated transitions over Earth's history have created the nested hierarchical structure we see today. In the following section, we give an overview of ETI theory.

In previous work, we developed a framework for K‐12 levels to teach the evolution of life's hierarchical structure using ETI theory (Hoskinson et al. 2024; Michod et al. 2022; Davison et al., n.d.). The present paper builds on the K‐12 curriculum to teach ETI theory and the evolution of hierarchical structure in university‐level courses. At K‐12 levels in the United States, there are national and state standards that teachers often use in designing their courses (see reference (NGSS Lead States 2013) and associated state standards). The closest analog to standards in post‐secondary biology education is the American Association for the Advancement of Science (AAAS) Vision and Change (AAAS 2011). Vision and Change was created by the AAAS to design effective and relevant university‐level biology curricula. However, most universities do not ask their instructors to align their course content to Vision and Change or other standards. Because there are no mandated standards for university‐level courses, we turn to college‐level biology and evolution textbooks in use in the United States to determine the topics likely to be taught at colleges and universities. Note that we use the terms “college” and “university” interchangeably in our paper.

Our paper is organized as follows. We first give an overview of ETI theory and its relationship to the theory of major evolutionary transitions, another framework used to describe different levels of the hierarchy of life. We then analyze the hierarchy of life as a topical area in university‐level introductory biology and evolution textbooks in common use in the United States. We next cover how to incorporate ETI theory into university evolution courses to teach the evolution of the hierarchy of life in two major sections. The first section focuses on how to expand upon and reframe the ETIs that are already being taught in current courses: the origin of life, the origin of the eukaryotic cell, the evolution of multicellularity, and the evolution of eusocial societies. The second section highlights how ETI theory can enhance or provide new perspectives on current topics already being taught in evolution courses. Finally, we discuss how teaching ETI theory can help instructors cover the elements of Vision and Change.

## 2 Overview of ETI Theory and Major Transitions

There are several overviews of ETI theory (Hanschen et al. 2018; Herron and Michod 2008; Michod 1999, 2007; Michod and Roze 1997; Michod and Herron 2006; West et al. 2015; Bourke 2011), including overviews with an education focus (Hoskinson et al. 2024; Michod et al. 2022; Davison et al., n.d.). Michod et al. (2022) describe three topical areas needed to teach ETI theory: (1) individuality as a core concept in biology, (2) cooperation and natural selection in social groups, and (3) the hierarchy of life. We have developed an instructional framework and curriculum sequence for teaching these concepts at K‐12 levels (Hoskinson et al. 2024) as well as tools for teaching these concepts at K‐12 levels (Davison et al., n.d.). The present paper builds on our K‐12 curriculum to teach these concepts in college courses. In doing so, we aim to address the major gap in college‐level curricula mentioned above and discussed further below: the disconnect between the hierarchy of life as an organizing principle for college‐level introductory biology courses and the limited coverage of the evolution of hierarchical organization in evolution courses.

During ETIs, groups of cooperating individuals evolve into a new kind of individual. This process is common to all ETIs and produces the nested hierarchical structure of evolutionary individuals we see today (i.e., genes, genomes, simple cells, complex cells, multicellular organisms, societies). Evolutionary individuals have the basic Darwinian properties of heritable variation in fitness. Consequently, ETI theory must explain how these Darwinian properties arise at the group level when initially they were present only at the individual level. To do this, ETI theory uses standard topics in evolutionary biology, including (1) multilevel selection involving gene frequency change in group‐structured populations (within‐ and between‐group selection), (2) the evolution of cooperation, conflict, and conflict mediation, (3) trade‐offs among fitness components, and (4) division of labor in fitness‐related traits subject to the aforementioned trade‐off.

An ETI involves the evolution of a group of individuals into a new kind of individual. ETI theory posits the following stages and selection pressures. The evolution of cooperation within groups allows for groups of individuals to carry out tasks that single individuals cannot. However, cheating is a fundamental problem for group living, and the evolution of conflict mediation mechanisms helps discourage cheating and enhance cooperation in the group. Conflict mediation mechanisms also drive the increase in heritability of fitness at the group level. Trade‐offs between fitness components (i.e., survival and reproduction) drive individuals to become specialized in the fitness components of the group. The specialization of individuals within the group leads to division of labor in fitness‐related traits in the group and selects for a commitment to group living. Division of labor in fitness components also prevents lower‐level individuals from reproducing and surviving independently, allowing for increased group integration and fitness heritability at the group level. The ongoing cycle of cooperation, conflict, and conflict mediation shapes the conversion of a group of individuals into a new kind of individual.

Since ETI theory is grounded in the same social principles that students experience in their lives (cooperation and conflict in social groups), we expect this approach to be engaging, intuitive, and likely to foster a deep understanding of evolutionary concepts. Social principles such as cooperation, cheating, and division of labor, which are commonly used to explain the behavior of human social groups, are also essential in explaining the behavior of biological individuals within a group. By drawing on these familiar principles, ETI theory provides a framework that students can relate to, making complex evolutionary concepts more accessible and relevant to students' own experiences.

In the evolutionary transitions literature, there are several different but overlapping frameworks, including “major evolutionary transitions” or MTEs and ETIs (Hanschen et al. 2017; Herron 2021). The development of the MTE framework goes back to the seminal paper of Maynard Smith (1988). Maynard Smith did not use the terminology “major” in this first paper on evolutionary transitions, nor did he consider changes in ways of transferring information as part of his framework. The notion of “major” and ways of transferring information are now components of MTEs (Maynard Smith and Szathmáry 1995), however, Maynard Smith (1988) was initially concerned with explaining evolutionary transitions between levels of complexity present in the hierarchy of life (see tab. 1 of Maynard Smith 1988). In other words, Maynard Smith (1988) was concerned with transitions in evolutionary individuality as used and understood in ETI theory to refer to the different kinds of increasingly complex evolutionary individuals present in the hierarchy of life. ETI theory is meant to address the original problem posed by Maynard Smith (1988) of explaining in Darwinian terms the evolution of the distinct levels of complexity present in the hierarchy of life.

ETI theory grew out of research into evolutionary individuality begun by Buss (1987) and Maynard Smith (1988) and subsequently developed by Maynard Smith and Szathmáry (1995) in their landmark book. The MTE framework (Maynard Smith and Szathmáry 1995; Szathmáry 2015) now includes both changes in evolutionary individuality (changes in the unit of selection and adaptation) and changes in the way in which information is transmitted (Maynard Smith and Szathmáry 1995; Szathmáry 2015). Consequently, MTEs are a mixture of different kinds of evolutionary events. For example, the evolution of the genetic code and the evolution of language are MTEs because they are major changes in the way in which information is transmitted. However, the evolution of the genetic code and human language are not changes in individuality; they are not changes in the unit or level of selection and adaptation. Therefore, the general MTE framework does not connect directly with the levels in the hierarchy of life. Using MTEs to explain the hierarchy of life is a step in the right direction, but it can be confusing because MTEs are a mixed collection of evolutionary events with different causes. In contrast, ETIs are a natural kind, a natural grouping of phenomena involving common problems and sharing common solutions (see, for example, Calcott and Sterelny 2011). We now turn to the role of hierarchical organization in college textbooks for introductory biology and evolution courses in the United States.

## 3 Textbook Analysis

### 3.1 Overview

We conducted a content analysis of four introductory biology textbooks (Urry et al. 2020; Mason et al. 2023; Freeman et al. 2024; Clark et al. 2020) and four evolution textbooks (Emlen and Zimmer 2020; Futuyma and Kirkpatrick 2023; Bergstrom and Dugatkin 2023; Herron and Freeman 2013) that are commonly used in undergraduate biology courses in the United States. Our selection of textbooks was based on our own teaching experience and input from colleagues. Collectively, we have taught across multiple departments and institutions for many years, providing a broad perspective on commonly adopted textbooks. Our content analysis of these textbooks focused on two primary queries: (1) the extent to which each textbook discussed the hierarchical organization of life and (2) the extent to which each textbook discussed the origin and evolution of the hierarchy of life (Table S1).

### 3.2 Methodology

Our content analysis used a deductive coding approach to address the two primary queries stated above; as such, we first co‐developed a codebook from which we could analyze themes across the introductory biology and evolution textbooks (see Table S2 for codebook). In the following paragraphs, we discuss the key elements that were included as codes that were to be applied to each of the textbooks.

**FIGURE 1:**
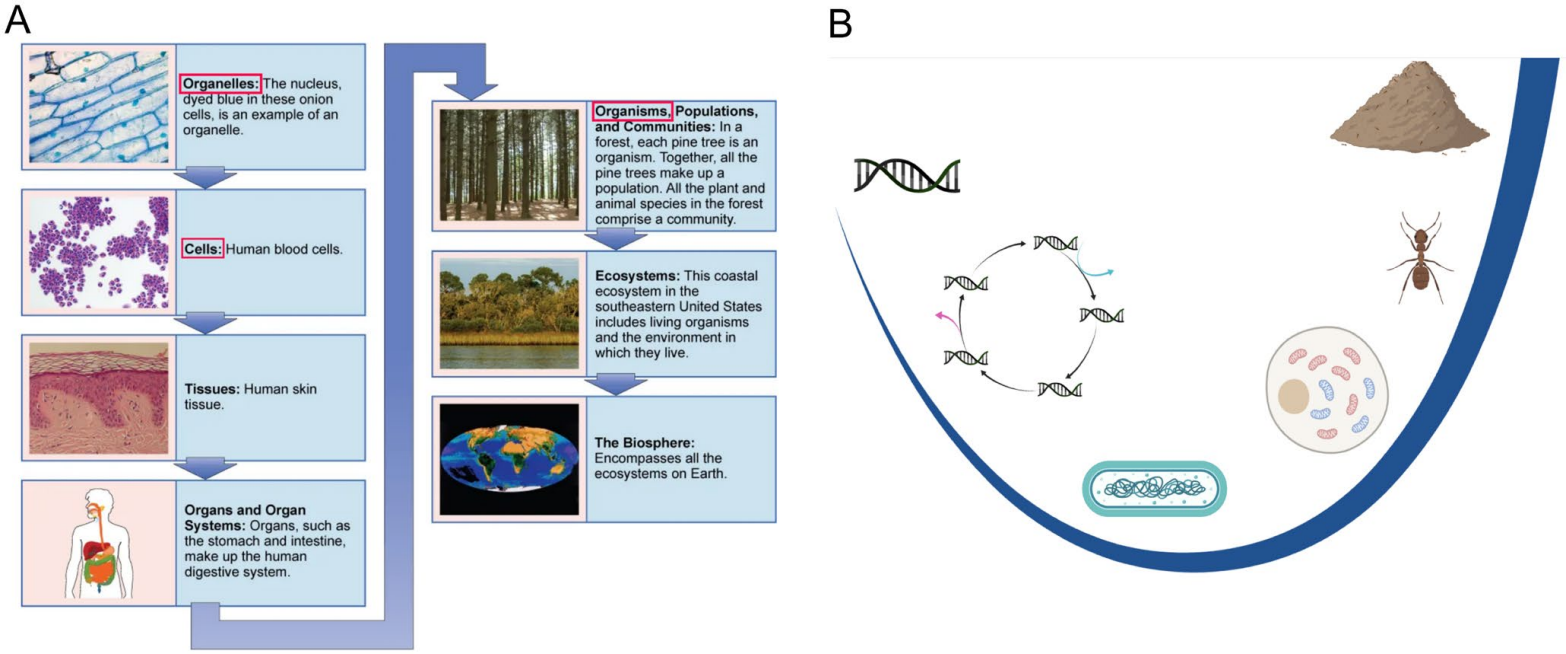
Example of the hierarchy of life display items found in analyzed textbooks. (A) Shows a typical hierarchy present in introductory biology textbooks reproduced from Clark et al. (2020), Creative Commons license v.4. Note, as is commonly the case, (A) includes both evolutionary and non‐evolutionary individuals; evolutionary individuals are highlighted with the pink boxes we have added. (B) Presents a hierarchy of life depicting only evolutionary individuals, reflecting a framework where each level emerges from ETIs: Groups of lower‐level individuals evolve into a new kind of individual (reproduced from Michod et al. (2022), with permission).

First, we examined whether the hierarchy of life is an important topic in the textbook by looking for display items and/or sections and/or the amount of text devoted to hierarchical organization. We analyzed the inclusion of display items, broadly defined as any means of visualization in the textbook, such as a figure or diagram, that addressed or presented the hierarchical organization of life (See Figure 1A for an example). Our analysis also considered whether these display items featured only evolutionary individuals (See Figure 1B for an example), in accordance with ETI theory, or if these display items featured both evolutionary individuals and non‐evolutionary individuals (as in Figure 1A). Additionally, we examined the extent to which each textbook emphasized the hierarchy of life by analyzing whether the textbook was broadly organized around the hierarchy of life and/or the amount of written material devoted to the hierarchy of life.

Second, we examined whether the *evolution* of the hierarchy of life was covered in each textbook. We considered whether the evolution of hierarchical organization was treated as a separate topic and/or whether the textbook covered the origin and evolution of particular levels in life's hierarchy (e.g., protocell, eukaryotic cell, multicellularity, and eusociality). If the evolution of one or more levels of the hierarchy of life was discussed, we identified which evolutionary framework (MTE or ETI, see discussion above) and evolutionary mechanisms were used to explain their evolution. If multiple levels of life's hierarchical structure were discussed in the textbook, such as, for example, multicellularity and the eukaryotic cell, we asked whether these levels were treated as separate topics or were they presented as connected, such as involving common evolutionary mechanisms or being levels in the hierarchy of life.

For the textbook analysis, the coding team (S.L., Z.I.G.‐W., J.S.H., D.R.D.) initially assigned textbooks for the purpose of preliminary review, which focused on combing through each textbook and identifying any and all relevant display items, section(s), and text relevant to the primary queries stated above. Textbook assignments were made based on the availability of each textbook to the coder, and each textbook had two different coders assigned to it. Once preliminary reviews were conducted, all coders met and reviewed each relevant display item, section(s), and text for each textbook. The coding team analyzed each display item, section(s), and text using the codebook developed above (see Table S2) as an entire group, and any disagreements were discussed and resolved with the entire coding team before a code was applied to the textbook. The codes assigned to each textbook as a result of the analysis are located in Table S1.

### 3.3 Results of Textbook Analysis

For introductory biology textbooks, our analysis revealed that the hierarchy of life is an important organizing framework, but the textbooks do not consider how and why the hierarchy of life evolved (Table S1). For example, Urry et al. (2020) and Mason et al. (2023) present the hierarchy of life as a display item at the beginning of the textbook and highlight it as a major theme or concept. However, the evolution of the hierarchy itself is not discussed in any of the four introductory biology textbooks. This is similar to the conclusion we reached for K‐12 biology curricula (Hoskinson et al. 2024; Michod et al. 2022). Although the evolution of certain levels of the hierarchy (i.e., eukaryotic cell, multicellularity, or social insects) is discussed in Urry et al. (2020) and Mason et al. (2023), these topics are scattered throughout the textbook without making a connection to the hierarchy of life itself. Mason, Losos, and Duncan (Urry et al. 2020) teach concepts necessary for understanding the evolution of life's hierarchy, such as cooperation and division of labor, but again, fail to make a connection to hierarchical organization. As a result, students have no reason to connect the evolution of the eukaryotic cell or multicellularity, distinct levels in the hierarchy of life, to each other and to the evolution of the hierarchy of life itself. It is not clear from the material presented in the text whether one level should relate to or be connected to another level in a nested hierarchical way.

The disconnect between hierarchical organization and evolution is further exemplified by the display item for the hierarchy of life itself (usually a figure) which includes *both* evolutionary levels (e.g., cells, multicellular organisms) and non‐evolutionary hierarchical levels (e.g., tissues, organs, ecosystems) without distinguishing which levels can be units of selection and adaptation, that is, which levels have evolved as evolutionary individuals (Figure 1).

A different pattern is present in evolution textbooks. Unlike in introductory biology textbooks, the hierarchy of life is not an important organizing principle in evolution textbooks and is not discussed as a self‐contained topic (Table S1). However, all four evolution textbooks cover particular levels of the hierarchy, and Bergstrom and Dugatkin (2023) dedicate a chapter to these levels, labeling them as MTEs. Furthermore, Bergstrom and Dugatkin (2023) and Futuyma and Kirkpatrick (2023) discuss evolutionary principles needed to explain the evolution of hierarchical organization (group formation, cooperation, division of labor) and Bergstrom and Dugatkin (2023) discuss individuality. Since introductory biology textbooks use the hierarchical structure of life as an organizing framework but do not teach its evolution, and evolution textbooks teach the evolutionary principles and separate transitions without connecting them to the hierarchy of life, there is a gap in instruction. This instructional gap could readily be filled by connecting common college‐level evolutionary biology content with the hierarchical organization through the teaching of ETIs.

As introductory biology textbooks use the hierarchy of life as an organizing framework but do not teach its evolution, there is a missed opportunity for evolution courses to pick up from where introductory biology courses leave off and emphasize and teach the evolution of hierarchical organization. Our recommendation is that the evolution of hierarchical organization be better emphasized in evolution courses so that students clearly see that evolutionary principles can explain the basic hierarchical scaffold on which biological diversity and complexity are organized. Significant advancements have been made in explaining the evolution of life's nested hierarchical structure and complexity in the last 50 years through the evolutionary transitions literature. ETI theory can address this disconnect by using commonly taught evolutionary principles in a generalized framework that connects the evolution of each level to life's nested hierarchical structure we observe today. We now turn to the topic of how to best teach hierarchical organization and ETIs in established evolution courses.

## 4 Using ETIs to Teach Hierarchical Organization in Evolution Courses

To bridge the gap in knowledge progression from introductory biology to evolution courses, we recommend teaching students about the evolution of hierarchical organization using ETIs. College‐level biology and evolution courses often include lectures dealing with the origin of life, the origin and evolution of the eukaryotic cell, and the evolution of multicellularity. These topics are sometimes present, if only briefly, in introductory biology courses. In this section, we illustrate how ETI theory and concepts can be used to teach these existing topics, each of which concerns the evolution of a key level in the hierarchy of life. While the details of these topics differ, they all involve the evolution of groups of individuals into a new kind of individual and therefore involve common principles of ETI theory, especially cooperation and conflict in groups. The same is true for the origin and evolution of eusocial societies in insects and mammals, although this transition is not discussed here in any detail.

Consider, first, the origin of life in terms of the evolution of a cooperating network of genes or a primitive genome in a protocell (Eigen 1971; Eigen and Schuster 1977; Szathmáry and Demeter 1987; Michod 1983). Initially, the evolutionary individuals are hypothesized to be RNA replicators (i.e., genes) that can survive and reproduce independently. These RNA replicators can form cooperative groups in which replicators support each other's survival and reproduction through replicase activity (Eigen's hypercycles). For example, consider a cooperative group of replicators (A and B) in which mutants can appear that increase or decrease cooperation. A more cooperative “altruistic” mutant of A gives more to B at a cost to itself. In contrast, a less cooperative “selfish” mutant of A will give less to B, while benefiting itself. In the absence of selection at the group level, selfish mutants are selected over altruistic mutants, and the cooperative network or primitive genome disappears. Encapsulating the cooperative network into a cellular compartment mediates this conflict, as the compartment aligns the evolutionary interests of the replicators inside and enhances selection at the group level. Groups with selfish mutants (now within a protocellular compartment) do worse than groups with more altruistic members, and selection at the group level can lead to the evolution of networks of cooperating genes or a primitive genome. During the transition to cellular life, the RNA replicators may further specialize in specific fitness components of the cell (survival or reproduction), resulting in a group where members cannot survive and reproduce on their own. This specialization reorganizes fitness components among the replicators and shifts fitness from being a property of the replicators to being a property of the group of replicators in a protocell. At the end of the transition, cooperating RNA replicators evolve into a genome in a simple cell, becoming a new level in life's hierarchical structure.

The same principles can be applied to teaching the evolution of the eukaryotic cell (Blackstone 2016; Michod and Nedelcu 2003a). The origin of the eukaryotic cell involves groups of genetically unrelated individuals, as is the case with the transition involving RNA replicators. The initial evolutionary individuals are prokaryotic cells, likely an archaeon and an alphaproteobacterium. Group formation may initially occur due to cross‐feeding and coupled metabolic pathways until the archaeon engulfs the alphaproteobacterium through endosymbiosis, which may lead to a tightly integrated symbiotic relationship. Multilevel selection and reciprocation can select for more cooperative endosymbionts. Conflict may arise when selfish endosymbionts increase their fitness at the expense of the host. This conflict is mediated by the evolution of mechanisms such as genomic reduction of the endosymbiont, which prevents the endosymbiont from reproducing and surviving by itself. This integration between host and symbiont metabolisms and genomes can transfer fitness to the group level. At the end of the transition, the symbiotic prokaryotic cells evolve into a new kind of cell, the eukaryotic cell, and become a new kind of individual, a new level in the hierarchical organization of life.

During the evolution of multicellularity, the initial evolutionary individuals are cells (Michod and Roze 2001). Group formation can occur either through the aggregation of separate cells or daughter cells staying together after cell division. Group membership may provide multiple benefits, such as buffering the internal environment from external environmental challenges (De Carpentier et al. 2019; Cheloni and Slaveykova 2021), avoiding predation (Boraas et al. 1998; Kirk 2003; Justice et al. 2008; Pérez et al. 2011), and increasing swimming speed (Short et al. 2006; Solari et al. 2006; Kuzdzal‐Fick et al. 2007). However, as the group size increases, selfish cells may appear whose reproduction benefits themselves at a cost to the group. The resulting conflict may be mediated through the division of labor and cellular specialization of fitness components (i.e., somatic and germ cells). This specialization and reorganization of fitness components shift fitness from being a property of cells to being a property of the group (Michod and Nedelcu 2003a, 2003b). Unlike the origin of hypercycles and the eukaryotic cell, the selection mechanics may involve kin selection and genetic relatedness among cells, especially if the cell group is formed through cells staying together after division. Hamilton's rule, which is often taught in undergraduate evolution courses, describes the condition for the spread of an altruistic gene that decreases the fitness of an individual but increases the fitness of genetic relatives who also carry the individual's genes (Hamilton 1963, 1964). Multilevel selection (another topic taught in undergraduate evolution courses) selects for cooperative groups that have a higher group fitness than a group with selfish cells. At the end of the transition, cooperating cells evolve into a multicellular organism and become a new level in the hierarchical organization of life.

## 5 Using ETIs to Enhance Common Topics in Evolution Courses

### 5.1 Overview of Section

In the previous section, we considered how ETI theory can use familiar topics in evolutionary biology, including the origin of life, the eukaryotic cell, and multicellularity, to explain the evolution of the hierarchy of life. Although not explicitly considered here, the evolution of eusociality in animals fits into the same framework (West et al. 2015; Bourke 2011). These familiar topics are a natural fit for teaching hierarchical complexity, as they are themselves levels in the hierarchy of life. If these topics are not being taught, instructors can still use ETI theory to connect to other key evolutionary biology topics discussed below. These topics likely occur in any curriculum on evolution, making them broadly accessible for teaching ETI theory internationally. We hope that instructors interested in incorporating ETIs into their existing courses will find ideas here on combining ETI concepts with their existing content.

### 5.2 What Is Life?

Many biology courses, both introductory biology and evolution courses, have a module or discussion about what life is. This content typically involves a discussion of multiple criteria for delineating life, including the criterion that life requires participation in Darwinian evolution. This is a natural point at which to introduce the concept of an individual as the entity that has Darwinian fitness, in that it both survives and reproduces. Evolutionary individuals also pass on their properties to offspring. Evolutionary individuals are levels of selection and adaptation. A module on the properties of life that incorporates individuality may help address student confusion surrounding biological organization and the distinction between parts and wholes. For example, students can compare a multicellular organism (e.g., a mammal) and a mammal's limb and ask a direct question: Which can both survive and reproduce? While the mammal's limb may fulfill criteria for identifying life, it cannot survive on its own and cannot reproduce itself, whereas the multicellular mammal as a whole can do both. Depending on context, cells may be individuals (like with bacteria) or parts of individuals (as in multicellular organisms), and evolutionary individuals may be molecular replicators, cells, multicellular organisms, etc. Additionally, the focus on evolutionary individuals as entities that have the Darwinian properties of survival and reproduction and are acted on by natural selection helps students make connections between the “what is life” content and the study of evolution.

### 5.3 Natural Selection

The unit of selection and adaptation is the evolutionary individual. To operationalize natural selection as changes in gene and genotype frequency over time, biologists count individuals. Although extensive literature on biological individuality exists (Calcott and Sterelny 2011; Van Baalen and Huneman 2014; Bouchard and Huneman 2013; Gisis et al. 2018), individuality itself is usually not discussed in textbooks (except for Bergstrom and Dugatkin 2023). It is common to teach evolution as gene frequency change, but it is often taken for granted what is to be counted to measure gene frequency. “Organisms” are counted, but this belies the complicated nature of what is an organism, which is a similar question to what constitutes an individual (Queller and Strassmann 2009). Including individuality as a topic forces this issue in a way that connects with standard course content, such as how to measure natural selection, and general properties of life, such as its hierarchical structure.

Individuality is a key concept for ETI theory because ETIs consider how a group of individuals evolves into a new kind of individual. Fitness must be transferred from the old to the new level. Teaching the concept of individuality can help students understand the connection between fitness, levels in the hierarchy of life, and the units with heritable variation in fitness that natural selection acts on. Furthermore, the multilevel nature of natural selection when populations are structured into groups and the tension between within‐group and between‐group selection can be taught using the evolution of cooperation and conflict, another key content area for ETIs. Between‐group selection favors the spread of cooperation and within‐group selection favors cheating, leading to a loss of cooperation. Conflict mediators (e.g., punishment and the germline) must evolve to overcome the temptation to cheat, if the group is to evolve into a new kind of individual. Expanding natural selection to include these topics and ETI theory explains how a group becomes a new kind of individual through selection acting on adaptations that allow for the division of labor and the reorganization of fitness components among individuals within a group.

### 5.4 Taxonomy, Phylogeny, and the Hierarchy of Life

Students typically learn about three hierarchical systems to help understand life's diversity: the Linnaean taxonomic system, phylogeny, and the hierarchy of life. These systems share the property of hierarchical nestedness, but they are different. The Linnaean system of naming species assigns organisms to a nested hierarchy of taxonomic ranks, from domain to species. Phylogenetic systematics groups organisms based on their evolutionary history, with monophyletic clades reflecting evolutionary relationships among species (Withgott 2000). In modern biology, Linnaean taxonomy should align with nested monophyletic groups from phylogenetic systematics, but due to historical use of names and lags between name changes and the generation of new phylogenies, this is not always the case. In contrast, the hierarchy of life classifies organisms by their level of organization as units of evolution and adaptation, independent of evolutionary history, as some transitions, like multicellularity, have occurred repeatedly across the history of life in both bacteria and eukaryotes (Grosberg and Strathmann 2007; Umen and Herron 2021).

ETI theory provides students with a framework to compare and contrast these classification systems. ETIs are the outcome of a natural selection process occurring in groups. By understanding ETIs, students can understand how evolutionary processes shaped the hierarchy of life. Phylogenetic trees, commonly used in research (Herron and Michod 2008; Lindsey et al. 2021, 2024) and teaching (Michod et al. 2022; Davison et al., n.d.), help bridge the gap between phylogenetic relationships and the hierarchical organization of life. Incorporating ETIs into the curriculum also helps students see the limits of Linnaean classification. ETIs emphasize the convergence of evolutionary units to create a new Darwinian unit of evolution and adaptation, whereas taxonomy is based on the progressive splitting of groups of organisms with shared characteristics.

### 5.5 Microevolution and Macroevolution

The distinction between micro‐ and macroevolution, and how microevolutionary changes may lead to macroevolutionary events, is a common topic in evolution (Emlen and Zimmer 2020; Futuyma and Kirkpatrick 2023; Herron and Freeman 2013) and introductory biology textbooks (Urry et al. 2020; Freeman et al. 2024; Clark et al. 2020). ETI theory explains how standard microevolutionary processes contribute to a major macroevolutionary event overlooked in textbooks—the creation of a new kind of evolutionary individual and level of organization in the hierarchy of life. The evolution of multicellularity in the Volvocine green algae, a macroevolutionary event, can be used to illustrate this. Microevolutionary events, such as gene duplication and co‐option of the *rls1* gene in unicellular *Chlamydomonas reinhardtii* to the *regA* gene in multicellular *Volvox carteri*, allowed for the development of somatic cells and division of labor that is the basis for the emergence of a new multicellular individual (a macroevolutionary event) (Grochau‐Wright et al. 2023). Thus, ETIs present a unique way to explicitly link the micro and macro views of evolution, which are often taught separately.

### 5.6 Evolution of Complexity

The evolution of complexity is often taught by focusing on single traits (such as the eye) to illustrate how a complex trait can evolve through small steps that are each advantageous in itself (Emlen and Zimmer 2020; Futuyma and Kirkpatrick 2023; Bergstrom and Dugatkin 2023; Herron and Freeman 2013). By teaching ETIs, instructors can also cover the evolution of hierarchical complexity. ETIs explain the jumps in complexity separating levels in the hierarchy of life. Complexity (measured by the number of parts, degree of integration, or hierarchical nestedness) has increased dramatically along certain branches of the tree of life. ETI theory explains these increases in complexity by explaining how groups become integrated and how fitness shifts from the level of the individual to the level of the group. None of the textbooks we examined explicitly addresses the evolution of the hierarchical organization of complexity and how repeated evolutionary transitions gave rise to the diversity of hierarchical complexity we see today. As mentioned, two textbooks discuss MTEs and include several examples of ETIs, although they do not connect these transitions to hierarchical complexity (Futuyma and Kirkpatrick 2023; Bergstrom and Dugatkin 2023).

When discussing the evolution of complexity, instructors must guard against the common but mistaken impression among students that complexity always increases during evolution. Beginning with the origin of life as simple replicators, there is no direction for complexity to go but to increase. What is interesting is the dramatic jumps in complexity that have occurred during the evolution of the hierarchy of life. Nevertheless, evolution is not a progressive march to increased complexity. Single‐celled organisms are still dominant on the planet today, and well‐identified reversions in complexity have been documented in many groups (O'Malley et al. 2016).

### 5.7 Evolution of Novelty

The evolution of novelty is usually taught in evolution courses through the evolution of novel phenotypic traits (i.e., innovations or exaptations that are modified ancestral structures with new functions shaped by natural selection) (Futuyma and Kirkpatrick 2023; Bergstrom and Dugatkin 2023). ETIs offer a new perspective with which to teach the evolution of “novelty” by explaining the evolution of novel kinds of evolutionary individuals, new levels of selection, and adaptation that exist in the hierarchy of life. ETI theory explains how a shift in selection can occur from an individual to a group of individuals through the evolution of novel traits (e.g., division of labor) and in the process create a new kind of evolutionary individual at a new hierarchical level.

### 5.8 Genome Evolution

As discussed above, ETI theory explains the origin of the genome itself as the transition from selfish genetic replicators to groups of cooperating replicators or primitive genomes in a protocell (Eigen 1971; Eigen and Schuster 1977; Szathmáry and Demeter 1987; Michod 1983). In addition, ETIs provide a framework to discuss aspects of eukaryotic genome evolution. For example, gene loss and genome reduction in organelles, horizontal gene transfer, and gene gain in the nucleus were necessary during eukaryotic cell evolution. According to the ETI theory, gene loss or transfer of fitness‐mediating genes from the endosymbiont to the host was necessary to prevent cheating and allow fitness to become a property of the cell group, the emerging eukaryotic cell. Increased developmental complexity is accompanied by genetic co‐option in the Volvocine green algae lineage along with gene loss, suggesting that the transition from unicellular to colonial or multicellular states resulted from gene network rewiring rather than the addition of new “multicellularity genes” (Jiménez‐Marín et al. 2023).

### 5.9 Evolution of Cancer

The evolution of cancer is often taught from a molecular perspective, focusing on genetic mutations that disrupt normal cell cycle regulation and evade tumor suppressor genes that result in uncontrolled cell growth (Urry et al. 2020; Mason et al. 2023; Freeman et al. 2024; Clark et al. 2020; Emlen and Zimmer 2020; Futuyma and Kirkpatrick 2023). ETI theory offers an evolutionary perspective of cancer, viewing it as a disruption of cellular cooperation within a multicellular organism. Cancer represents a reversal of an ETI, where cells revert to a unicellular state through cheating by engaging in unregulated division, thereby disrupting key features of multicellularity (Aktipis et al. 2015).

## 6 Alignment of ETIs to the AAAS “Vision and Change”

Vision and Change is a call to action and summary report based on meetings and discussions of over 500 biology faculty from 2‐ and 4‐year colleges and universities, organized by the AAAS (2011). Vision and Change provides a broad framework to build upon when designing effective and relevant modern biology curricula. Indeed, Vision and Change has inspired multiple innovations in organizing undergraduate curricula (Brownell et al. 2014; Momsen et al. 2022), assessing student learning (Couch et al. 2019; Smith et al. 2019), and guiding education research (Tripp and Shortlidge 2019; Aikens 2020).

Vision and Change describes five core concepts all undergraduates studying biology should learn and understand: (1) evolution; (2) structure and function; (3) information flow, exchange, and storage; (4) pathways and transformations of energy and matter; and (5) systems thinking that “focuses on emergent properties at all levels of organization, from molecules to ecosystems to social systems.” Understanding biological systems and hierarchical organization is a recurring theme that is emphasized across all core concepts. For example, the document states, “A strong preparation in the theory of evolution remains essential to understanding biological systems at all levels” or “All students should understand that all levels of biological organization depend on specific interactions and information transfer” (Davison et al., n.d.).

ETI theory aligns with and provides new perspectives to teach the Vision and Change core concepts to undergraduate students. The hierarchical organization of life is a central theme in introductory biology textbooks and is the basis of a core concept of Vision and Change, but this topic is generally missing from evolutionary biology textbooks. ETI theory provides an explanatory framework for how the hierarchy of life evolved through evolutionary processes. Teaching students about ETIs can also encourage them to think critically about the units of natural selection and how those units can change during evolution. Although we align ETI theory with Vision and Change, our framework is internationally applicable, as it was originally developed based on NGSS, which was benchmarked against international teaching practices and standards (NGSS 2010).

The core concepts of structure and function, and information flow, exchange, and storage can be taught through examining the evolution of conflict mediation mechanisms. For example, germ–soma cellular differentiation is an important conflict mediation mechanism shaping the evolution of multicellularity. Different cell types evolve through structural modifications that support the functional role they play for the multicellular group. Moreover, novel information flow and exchange pathways, such as cell–cell communication mechanisms, must evolve to regulate cellular differentiation. Similarly, through germ–soma cell differentiation and high kinship among cells (assuming the evolution of multicellularity in clonal groups), the flow of information from one generation to the next changes as well: from cell to cell to multicellular organism to reproductive cell to multicellular organism.

ETI theory also adds an evolutionary angle to the study of pathways and transformations of energy and matter, a core concept in two ways. First, ETIs were at the center of two of the greatest energy revolutions in the history of eukaryotes: the evolution of aerobic respiration and oxygenic photosynthesis through the endosymbiosis of mitochondria and chloroplasts, respectively. Teaching these events through an ETI lens shows that they are part of a broader set of evolutionary processes rather than idiosyncratic singular events in Earth's history. Second, during an ETI, new pathways for sharing resources and energy within a group must evolve. For example, the evolution of multicellularity is associated with the evolution of increased group/body size and cellular differentiation. As a multicellular group increases in size and cells specialize, novel geometries or nutrient/energy exchange pathways must evolve (such as circulatory systems) so that all cells can receive sufficient nutrients and excrete wastes without overexploiting other cells. Even in cell groups without circulatory systems, like the Volvocine green algae, flagellar beating can break down boundary layers at the edge of the group and increase the flow of nutrients and waste products in and out of the group, respectively (Short et al. 2006; Michod et al. 2006; Solari et al. 2015).

Finally, ETI theory provides a useful framework to teach undergraduates about biological systems and systems thinking. The Vision and Change framework recognizes that “A systems approach to biological phenomena focuses on emergent properties at all levels of organization, from molecules to ecosystems to social systems (AAAS 2011).” ETI theory provides a framework for explaining how key levels of organization arise in biological systems and how fitness and group‐level functions emerge at the group level through evolutionary processes, including group formation, conflict, conflict mediation, and integration among group members to form a new kind of organism at a higher level of hierarchical organization.

Vision and Change also describes core competencies and disciplinary practices that all undergraduates learning biology should develop. These core competencies include the ability to (1) apply the process of science, (2) use quantitative reasoning, (3) use modeling and simulation, (4) tap into the interdisciplinary nature of science, (5) communicate and collaborate with other disciplines, and (6) understand the relationship between science and society. As with the disciplinary core concepts, incorporating the ETI framework into introductory biology courses can provide engaging ways for students to develop the Vision and Change core competencies.

ETI theory was developed using mathematical models (population genetics, optimization theory, and game theory). Working through examples and practice problems of cooperation and conflict mediating mechanisms can allow students to use quantitative reasoning and modeling to test hypotheses about when high group cooperation will and will not evolve. Cooperation models such as the Prisoner's Dilemma are also used in a wide range of disciplines, including economics, psychology, and political science, which can illustrate how concepts taught in biology courses have interdisciplinary and societal implications and relations (Snyder 1971; Jervis 1978; Axelrod 1980; Holt and Capra 2000). Similarly, ETIs involve phenomena that span disciplinary studies. For example, the study of cellular differentiation integrates cell, molecular, and developmental biology (Kirk 2005; Brunet and King 2017), investigating the timing and paleoecological context of ancient ETIs involves paleontology and geology (Lindsey et al. 2024; Dacks et al. 2016), and research on the effects of increased size on organismal biology draws on physics and hydrodynamics (Solari et al. 2006). Lastly, the social principles used to explain ETIs apply to groups of humans and form the fabric of human society, bridging evolutionary biology with fields such as sociology and psychology. Studying ETIs may also enhance understanding of the relationship between science and society by using concepts of cooperation and conflict to show how evolutionary science relates to social structures and ethical issues. Thus, ETI theory provides an evolutionary lens that may be applied to teach or reinforce all of the Vision and Change core competencies and core concepts.

## 7 Discussion

### 7.1 Why Teach ETIs?

The hierarchy of life is a fundamental feature of the living world. It was not present at the origin of life, it evolved, and students should understand how and why. The disconnect between introductory biology's emphasis on the hierarchy of life as an organizing framework and how this hierarchical organization evolved can be bridged by teaching ETIs. Textbook analysis of four introductory biology and four evolution textbooks revealed that the hierarchy of life is presented as an organizing framework in introductory biology but evolution textbooks do not use this framework or teach its evolution. Consequently, students do not learn how it evolved and are left to wonder if evolution can explain one of life's most general and familiar properties. The teaching of ETIs provides an evolutionary framework to understand the evolution of the hierarchical organization of life. ETI theory explains how groups of cooperating individuals evolve into a new kind of evolutionary individual. This evolutionary conversion of groups of existing individuals into a new kind of individual repeated during life's history is precisely what gives life its nested hierarchical structure.

Beyond explaining the evolution of the hierarchy of life, ETI theory offers additional benefits. We have discussed how ETI theory and the required key concepts enhance the teaching of various topics likely present in the evolution curriculum. By integrating ETI concepts into existing content, instructors can help students make connections between concepts, especially if they already cover different levels of the hierarchy of life, such as the origin of life in terms of cooperative genetic networks in a protocell, what is life, eukaryogenesis, multicellularity, or eusocial societies. ETI theory employs an intuitive social framework akin to real‐life situations students have experienced, which may lead to increased student engagement and a deeper understanding of evolutionary biology concepts.

We have discussed how teaching ETI theory offers a new approach to teaching the key concepts and competencies of Vision and Change (Davison et al., n.d.). For example, systems thinking is a key concept of Vision and Change, but previously suggested approaches in K‐12 biology education (systems biology or complexity science) (Kirk 2005; Wilson 2019; Hawley et al. 2011; Nadelson and Southerland 2012) take the system as given and do not explain how complex hierarchically organized systems evolve in the first place. Although “systems biology” is covered in the textbooks we considered (Urry et al. 2020; Mason et al. 2023; Freeman et al. 2024; Clark et al. 2020) and specifically called out for teaching in Vision and Change, the “system” is again specified, and its evolution is not considered. ETIs can bridge this gap by teaching the evolution of increasingly integrated cooperative systems, where members evolve specific roles, resulting in the system transitioning to a new level of hierarchical organization. By using ETIs to teach systems thinking, students can develop additional Vision and Change concepts, such as understanding how certain structures contribute to the individuality of the whole, how the evolution of these structures enables the evolution of novel pathways for information exchange and transmission as well as shifts in energy and matter transformations. This will likely enhance students' comprehension of complex biological systems as analogous to the social systems with which they are already familiar. Furthermore, ETIs provide engaging ways for students to develop the Vision and Change core competencies, such as understanding how science and society interconnect. Not only is biology conducted in a social context as Vision and Change observes, but biology itself, in its hierarchical structure, evolved out of social principles.

### 7.2 Entry Points in Existing Courses

How can instructors start teaching ETIs and the hierarchy of life in their existing courses? We can relate to instructors who already have too much material to teach in courses that are already packed with content. For this reason, we have indicated existing topics that can be augmented or enhanced by using ETI theory and concepts to teach the hierarchy of life.

We recommend that instructors first introduce “individuality” when discussing what life is, a common topic in most biology courses, introductory or advanced. Evolutionary individuals have the Darwinian properties of survival and reproduction and participate in the process of natural selection. Instructors may then pick up on the idea of individuality later when discussing the mechanics of natural selection and the need to count individuals to measure gene frequency. In addition to providing additional content on individuality itself, there is a need to instruct on the evolution of cooperation among individuals in groups and the integration of groups through division of labor and specialization in fitness components of the group. This (perhaps new) content includes selection mechanics and the integration of groups through multilevel selection and kin selection (if groups are formed through clonal development). Multilevel selection and the selection mechanisms underlying the evolution of cooperation are perhaps the most challenging new topics to include. In introductory courses, instructors can simply speak of the integration of groups into new kinds of individuals without delving into the selection mechanics (multilevel selection, kin selection, reciprocation) that cause this transition. The selection mechanics involving cooperation in groups can be picked up in more detail in evolution courses.

### 7.3 Challenges and Future Directions

The need for the teaching interventions proposed here has been theory‐driven; we have identified a gap in instruction concerning the evolution of the hierarchy of life and translated ETI theory from the scientific literature into an instructional framework that can be used to explicitly teach the evolution of the hierarchy of life (Hoskinson et al. 2024; Michod et al. 2022; Davison et al., n.d.). In addition, we have aligned our work to the NGSS (NGSS Lead States 2013) and the Vision and Change guiding framework (AAAS 2011) to maximize the utility and continuity of our instructional framework in primary, secondary, and post‐secondary levels of education. Future research will be focused on how our instructional frameworks impact student learning regarding evolutionary biology.

There are two key avenues for investigating the impact in biology education of teaching the evolution of hierarchical organization using ETI theory. First, as we have emphasized, because ETI theory is based on the same social principles that students encounter in their lives, we expect this approach to teaching biological complexity to be engaging and intuitive. Future research will investigate relationships between the teaching of ETIs and student engagement in introductory biology and evolution courses. Second, future research should focus on the effectiveness of the interventions proposed here for some of the long‐standing issues in teaching evolution, such as evolution acceptance. Published surveys suggest that a student's capacity to explain complexity affects their acceptance of evolution (Hawley et al. 2011; Nadelson and Southerland 2012; Short and Hawley 2012). Hoskinson et al. (2024) explain that existing curricula, including the NGSS, do not teach the evolution of hierarchical complexity. Therefore, we expect that incorporating instruction on the origin and evolution of biological complexity through hierarchical organization should positively impact evolution acceptance. We are creating lesson plans at various grade levels based on ETI concepts. We are also in the process of assessing whether implementing the interventions proposed here enhances students' understanding and acceptance of evolution.

## Author Contributions

**SoRi La:** conceptualization (equal), investigation (equal), methodology (equal), writing – original draft (equal), writing – review and editing (equal). **Zachariah I. Grochau‐Wright:** conceptualization (equal), investigation (equal), writing – original draft (equal), writing – review and editing (equal). **Joshua S. Hoskinson:** conceptualization (equal), investigation (equal), methodology (equal), writing – review and editing (equal). **Dinah R. Davison:** investigation (equal), writing – review and editing (equal). **Richard E. Michod:** conceptualization (equal), supervision (equal), methodology (equal), writing – original draft (equal), writing – review and editing (equal).

## Conflicts of Interest

The authors declare no conflicts of interest.

## Supporting information


**Table S1:** ece372267‐sup‐0001‐TableS1.docx.

**Table S2:** ece372267‐sup‐0002‐TableS2.docx.

## Data Availability

Collected data is provided as Supporting Information.
