# Supplementary material for: Translating Research on Evolutionary Transitions Into the Teaching of Hierarchical Complexity in University Biology Courses
Source: Ecol Evol. 2025 Nov 2;15(11):e72267. doi: 10.1002/ece3.72267 (PMC12580225; doi:10.1002/ece3.72267)
Supplement: Supplementary file 1 — Table S1: ece372267‐sup‐0001‐TableS1.docx. [file ECE3-15-e72267-s001.docx]

# S1 Table. Analysis of introductory biology and evolution textbooks on the use and explanation of the origin and evolution of the hierarchy of life.

|  |  | **Q1 Does the textbook consider the hierarchy of life as an important topic?** | | **Q2 Does the textbook discuss how the hierarchy of life evolved?** | | |
| --- | --- | --- | --- | --- | --- | --- |
|  |  | **Q1.1 Is there a display item describing the hierarchy of life? *Choose one option** | **Q1.2 To what extent is the hierarchical organization of life present in the textbook? *Choose all that apply** | **Q2.1 What framework is used to explain the evolution of the hierarchical organization? *Choose one option** | **Q2.2 Which particular levels of the hierarchy does the textbook explain its origin through Darwinian evolution? *Choose all that apply** | **Q2.3 What are the evolutionary mechanisms used to explain the evolution of the hierarchy of life or a specific level of the hierarchy of life? *Choose all that apply** |
| **Introductory biology textbook** | [**OpenStax Biology 2e**](https://bio.libretexts.org/Bookshelves/Introductory_and_General_Biology) **Edition**  **by Clark** | Display item with evolutionary and non-evolutionary individuals | Dedicated section/subsection/subunit | None | eukaryotic cell, multicellularity, eusociality | Cooperation, division of labor, selection and adaptation |
|  | **Biological Science 8^th^ Edition by Freeman et al.,** | Display item with evolutionary and non-evolutionary individuals | Less than a section | None | Protocells, eukaryotic cell, multicellularity, eusociality | division of labor, cooperation, selection and adaptation |
|  | **Campbell Biology 12^th^ Edition by Urry et al.,** | Display item with evolutionary and non-evolutionary individuals | Dedicated section/subsection/subunit | None | Protocells, eukaryotic cell, Multicellularity | Cooperation, selection and adaptation |
|  | **Biology 13^th^ Edition by Raven et al.,** | Display item with evolutionary and non-evolutionary individuals | Dedicated section/subsection/subunit | None | eukaryotic cell, multicellularity, Eusociality | division of labor, kin selection, cooperation |
| **Evolution textbook** | **Evolution 3^rd^ Edition by Bergstrom, Dugatkin, and Alan** | Display item (list) with evolutionary and non-evolutionary individuals | Dedicated section on MTEs | MTEs | Protocells, eukaryotic cell, multicellularity, eusociality, evolution of individuality | cooperation, selection and adaptation, conflict mediation, division of labor, multilevel selection, kin selection. |
|  | **Evolutionary Analysis 5^th^ Edition by Herron and Freeman** | No display item | Not present | None | Eusociality | Selection and adaptation, multilevel selection, cooperation, kin selection, division of labor |
|  | **Making sense of life 3^rd^ Edition by Emlen and Zimmer** | No display item | Not present | None | eukaryotic cell, multicellularity, eusociality | none |
|  | **Evolution 5^th^ Edition by** **Futuyma and Kirkpatrick** | Display item (table) of evolutionary individuals | Dedicated section on MTEs | MTEs | Protocells, eukaryotic cell, multicellularity, eusociality | cooperation, selection and adaptation, conflict mediation, division of labor, kin selection, multilevel selection |
