# Supplementary material for: Translating Research on Evolutionary Transitions Into the Teaching of Hierarchical Complexity in University Biology Courses
Source: Ecol Evol. 2025 Nov 2;15(11):e72267. doi: 10.1002/ece3.72267 (PMC12580225; doi:10.1002/ece3.72267)
Supplement: Supplementary file 2 — Table S2: ece372267‐sup‐0002‐TableS2.docx. [file ECE3-15-e72267-s002.docx]

S2 Table. Coding categories for the analysis of introductory biology and evolution textbooks on the use and explanation of the origin and evolution of the hierarchy of life.

| **Code** | **Definition** |
| --- | --- |
| Q1 Does the textbook consider the hierarchy of life as an important topic? | |
| Q1.1 Is there a **display item** describing the hierarchy of life? *choose one option | |
| No display item | *Display items are any means of visualization of the hierarchical organization of life, including figures, tables, and other diagrams.*  no display item exhibiting the hierarchical structure exists anywhere in the textbook. |
| Display item with **ONLY** evolutionary individuals | *Evolutionary individuals are biological entities that can survive and reproduce on their own (i.e., biological entities with heritable variation in fitness). Examples of evolutionary individuals include gene, protocell, cell, eukaryotic cell, prokaryotic cell, multicellular organism, and eusocial societies.*  The textbook includes a figure /diagram/table/list depicting the hierarchical organization of life with ONLY evolutionary individuals included. |
| Display item with evolutionary **AND** non-evolutionary individuals | *Evolutionary individuals are biological entities that can survive and reproduce on their own (i.e., biological entities with heritable variation in fitness). Examples of evolutionary individuals include gene, protocell, cell, eukaryotic cell, prokaryotic cell, multicellular organism, and eusocial societies.*  *Non-evolutionary individuals are biological entities that did not, at any point in the history of life on Earth, survive and reproduce on their own. This means that these biological entities did not, at any point in the history of life, have the characteristic of heritable variation in fitness. Examples of non-evolutionary individuals include tissues, organs, organ systems, populations, communities, and ecosystems.*  The textbook includes a figure /diagram/table/list depicting the hierarchical organization of life with evolutionary individuals **AND** non-evolutionary individuals included. |
| Q1.2 To what extent is the hierarchical organization of life present in the textbook? *Choose all that apply | |
| Not Present | hierarchy of life is not mentioned in the textbook |
| table of contents | Table of contents is organized according to the different levels of the hierarchy of life. This includes a general progression of topics presented in the textbook in the following general order: genes, cells, tissues, organs, organisms, and ecosystems. Chapters do not have to go in this exact order, but there is a clear sense that the textbook is organized using the hierarchy of life. |
| Dedicated major theme | Hierarchy of life is listed as a major theme in the textbook within the table of contents of the textbook. |
| Dedicated chapter/unit | There is a dedicated chapter discussing the hierarchy of life with a chapter heading included within the table of contents of the textbook. |
| Dedicated section/subsection/subunit | There is a dedicated section/subsection/subunit discussing the hierarchy of life with a section or subsection heading included within the table of contents of the textbook. |
| Less than a section | There is a dedicated paragraph or less discussing the hierarchy of life, however, there is no section or subsection heading included within the table of contents of the textbook. |
| Q2 Does the section discuss the hierarchy of life from an evolutionary lens? | |
| Q2.1 What **framework** is used to explain the evolution of the hierarchical organization? *Choose one option | |
| None | No evolutionary framework is mentioned |
| Evolutionary transitions in individuality | Evolution of the hierarchy of life is discussed as a transition involving a group becoming an evolutionary individual that is a new level of selection through fitness reorganization (i.e., Evolutionary transitions in individuality theory or ETI theory) OR one or more examples of ETIs are mentioned and explained in the textbook. Examples include genome, transposons, sex, protocell, chromosomes, eukaryotic cell, multicellularity, eusociality, sex, plastids, symbiosis |
| Major transitions (MTEs) | Evolution of the hierarchy of life is discussed as a change in the unit of selection and transfer of information (i.e., major transitions) OR one or more examples of major transitions are mentioned and explained in the textbook. Examples include genetic code, protocell, chromosomes, eukaryotic cell, multicellularity, eusociality, sex, language |
| Q2.2 Which particular levels of the hierarchy does the textbook explain its origin through Darwinian evolution? *Choose all that apply | |
| None |  |
| Protocells | Conflict mediation mechanism required for the transition from RNA replicators to cooperative gene networks/hypercycles |
| Eukaryotic cell | Formation of a group between the archaeal host and the alphaproteobacteria that transitioned into a eukaryotic cell with a mitochondrion |
| Multicellularity | Formation of a group of unicellular organisms that transitioned into a multicellular organism |
| Eusociality | Formation of a group of multicellular organisms that transitioned into a eusocial group |
| Q2.3 What are the **evolutionary mechanisms** used to explain the evolution of the hierarchy of life or a level of the hierarchy of life? *Choose all that apply *The codes must be considered within the context of the hierarchy of life. | |
| Not applicable | No explanation for the evolution of the hierarchy of life is given. |
| Selection and adaptation | Natural selection and adaptation drive the evolution of the hierarchical organization of life |
| cooperation | Interaction where individuals form groups and work together for mutual or one-sided benefit |
| Conflict mediation | Mechanism that aligns interests of individuals within a group to prevent cheating |
| Game theory | A mathematical framework used to model strategic interactions between individuals. |
| Division of labor | Individuals within a group specialize in a specific component of fitness |
| Multilevel selection | selection operates at multiple levels, such as within individuals and groups. This process contributes to the transfer of fitness from individuals to groups |
| Kin selection | Selection that decreases the fitness of an altruistic individual but increases the fitness of relatives within the group who also carry the individual’s genes |
